# Quantification of Nanoscale Dose Enhancement in Gold Nanoparticle-Aided External Photon Beam Radiotherapy

**DOI:** 10.3390/cancers14092167

**Published:** 2022-04-26

**Authors:** Elena Vlastou, Evaggelos Pantelis, Efstathios P. Efstathopoulos, Pantelis Karaiskos, Vasileios Kouloulias, Kalliopi Platoni

**Affiliations:** 1 Medical Physics Unit, 2nd Department of Radiology, University General Hospital “Attikon”, School of Medicine, National and Kapodistrian University of Athens, 12462 Athens, Greece; elenabls@med.uoa.gr (E.V.); stathise@med.uoa.gr (E.P.E.); vkouloul@med.uoa.gr (V.K.); 2 Medical Physics Laboratory, School of Medicine, National and Kapodistrian University of Athens, 11527 Athens, Greece; vpantelis@med.uoa.gr (E.P.); pkaraisk@med.uoa.gr (P.K.)

**Keywords:** radiotherapy, gold nanoparticles, dose enhancement, Monte Carlo simulations, nanodosimetry

## Abstract

**Simple Summary:**

Gold nanoparticles (AuNPs) have become common in radiation oncology research through the past decades. Their radiosensitization effect could offer a novel approach to cancer diagnosis and radiotherapy as well. The aim of this study is the assessment of dose enhancement attributed to the incubation of AuNPs in an irradiated target. The present work is focused on investigating the impact of AuNPs properties in dose increase under different irradiation conditions using 6 MV photon beams with and without a flattening filter via Monte Carlo simulations. Results from the simulated scenarios depict a sufficient dose raise especially at close distances to AuNPs, depending on the presence of a flattening filter in the path of the photon beam, AuNPs size, the type of modeled distribution and their concentration. Therefore, the obtained enhanced dose deposition due to AuNPs presence in an irradiated region could lead to more sufficient tumor cell destruction than irradiation alone.

**Abstract:**

The recent progress in Nanotechnology has introduced Gold Nanoparticles (AuNPs) as promising radiosensitizing agents in radiation oncology. This work aims to estimate dose enhancement due to the presence of AuNPs inside an irradiated water region through Monte Carlo calculations. The GATE platform was used to simulate 6 MV photon histories generated from a TrueBeam^®^ linear accelerator with and without a Flattening Filter (FF) and model AuNPs clusters. The AuNPs size, concentration and distribution pattern were examined. To investigate different clinical irradiation conditions, the effect of field size, presence of FF and placement of AuNPs in water were evaluated. The range of Dose Enhancement Factors (DEF = Dose_Au_/Dose_Water_) calculated in this study is 0.99 ± 0.01–1.26 ± 0.02 depending on photon beam quality, distance from AuNPs surface, AuNPs size and concentration and pattern of distribution. The highest DEF is reported for irradiation using un-flattened photon beams and at close distances from AuNPs. The obtained findings suggest that dose deposition could be increased in regions that represent whole cells or subcellular targets (mitochondria, cell nucleus, etc.). Nevertheless, further and consistent research is needed in order to make a step toward AuNP-aided radiotherapy in clinical practice.

## 1. Introduction

Gold nanoparticle (AuNP)-enhanced radiation therapy (RT) has attracted interest in radiation oncology over the last years [1,2,3,4,5,6,7]. This is due to the reported dose enhancement (defined as the ratio of the dose deposited in a voxel of water with and without the presence of AuNPs) [3,4] and tumor cell-killing increase [1,2,6,7,8] when a tumor region loaded with AuNPs is irradiated with a photon or a particle beam. 

The existing evidence supports that the greatest levels of dose enhancement or killing of tumor cells can be achieved using low kV photon beams [9,10,11]. These findings are consistent with the physical background underlying the interactions of photons with AuNPs. The difference in atomic number (Z) of gold and soft tissue along with the proportionality of photoelectric interaction probability to ~(Z/E)^3^ could be exploited for photons in the kV region. The cascade of secondary photons and short-range e^–^ (i.e., Auger e^–^) resulting from kV photon photoelectric interactions with gold could lead to an increase in dose deposition around AuNPs, which will, in turn, lead to more effective malignant cell destruction. In the MV range though, Compton and pair production are the main phenomena that describe photon interactions. Since Compton cross section has a weaker dependence on Z, it is expected that irradiation of tissues containing AuNPs using 6 MV (typical beam energy in external beam RT-EBRT) photon beams would lead to low dose enhancement. 

EBRT is delivered using a linear accelerator (linac). A flattening filter (FF)is mounted on the linac’s head and it is designed to generate uniform beam intensity over a photon field leading to homogeneous dose distributions in a tumor volume. However, modern RT techniques (e.g., Intensity Modulated RT-IMRT) can create conformal dose distributions in tumors, modulating dose intensity through numerous multileaf collimator-shaped subfields. Thus, FF can be removed from linacs leading to increased dose rates, decreased head scatter radiation and softer photon spectra [12]. FF-free (FFF) photon beams contain a higher proportion of low-energy photons compared to flattened ones, indicating a possible enhancement in photoelectric interactions of photons with AuNPs.

While Monte Carlo (MC) studies report controversial results concerning dose enhancement attributed to AuNPs presence in a target irradiated by photons in the MV region [4,9,10,11,13,14,15,16,17,18], a substantial radiosensitivity enhancement is described in vivo [7,19] and in vitro [6,20,21,22,23]. Most in silico studies predict a dose enhancement in the MV region ranging from 1% to 10% for 2–200 nm AuNPs and concentrations of 7–36 mg/g [10,11,14,15,16,17], while limited simulations report higher dose enhancement levels. In this context, Douglass et al. [9] calculated a dose enhancement ranging from 32% to 156%. In another study, where dose enhancement was reported as field size, depth and delivery mode dependent, an up to ~4.5-fold increase in dose was observed [13]. Tsiamas et al. [4] calculated dose enhancement for different clinical scenarios using a modeled MV linac. Dose enhancement values of the order of 4.7 and 17, were reported for in, and out of, photon field regions, respectively. It is noted though that regarding in silico approaches, the choice between track structure (TS) and condensed history (CH) simulation codes, the low energy detection limits, along with AuNPs, distribution modeling appears to be of critical importance for the precise quantification of AuNPs radiosensitization effect [24,25,26].

The majority of experiments in small animals and cell lines using MV photon beams report more promising results than most MC predictions. In more detail, Anijdan et al. [19] obtained dose enhancement values of 8–10% having performed irradiation with an 18 MV photon beam in mice with AuNP-loaded melanoma cells of 50 nm at a concentration of 7 mg/mL. Amani et al. [27] examined HeLa cells irradiation. Cells, containing 15 μg/mL nanospheres and nanorods, were exposed to 6 MV photons and their viability was found to be 40% and 20%, respectively, when the corresponding value without AuNPs was about 85%. Hau et al. [21] irradiated Polyethylene Glycol (PEG) AuNP-loaded (50 μg/mL) human colon cancer cells with 6 MV photons and measured a 35.3% increase in their cytotoxicity. A meaningful sensitization enhancement of 1.45 on glioblastoma cells in the presence of 42 nm AuNPs irradiated with a clinical 6 MV photon beam in a concentration of 100 µg/mL was also recently reported by Kazmi et al. [22]. Zhang et al. [23] examined the radiosensitization in colorectal cancer cell line after incubation of 400 nM GNP-PEG-R8 and found a sensitivity enhancement ratio of 1.59. Beam quality, AuNPs concentration and their physicochemical characteristics, the method of AuNPs administration and the type of cell line investigated are only some of the parameters that appear to influence dose enhancement in published in vivo and in vitro studies [6,27,28,29].

Considering the huge diversity among simulation strategies and experimental protocols, comparing findings between different studies could be misleading. Moreover, the discrepancies between in silico and in vitro/in vivo experiments complicate the implementation of AuNPs in clinical EBRT and underline the need for further and consistent research. Since the greatest increase in dose deposition has been reported at close distances to AuNPs [4,5], microscopic dosimetric approaches in silico are likely to reveal dose increase in cell regions and match better with the in vitro findings. In this work, dose deposition was estimated inside a water phantom for different photon beam configurations generated from a 6 MV linac as a function of distance from AuNPs clusters, through MC calculations. As for AuNPs, their size, concentration and type of distribution were examined. Aiming to investigate different clinical irradiation conditions, the effect of field size, presence or absence of FF and placement of AuNPs within the water on dose enhancement was evaluated. The upper goal of the present study is the quantification of dose enhancement in endothelial cells (~2 μm), typical cells (~10 μm), and regions that could include subcellular radiation targets such as mitochondria (0.5–1 μm) and cell nucleus (~1 μm), attributed to MV photons irradiation of AuNPs cluster models. 

## 2. Materials and Methods

### 2.1. Simulation Details

All simulations were performed using GATE 8.2 (GEANT4 application for tomographic emission) [30]. The Emstandard_opt3 electromagnetic physic list was used in order to simulate electromagnetic processes (valid for energies 1 keV–100 TeV) in terms of photon beams modeling. Compared to CH physics models, which consider the effect of a large number of interactions along a “step”, TS physics models are more suitable to describe nanoscale electron transport in water since they enable simulation of charged particles interactions event-by-event down to ~eV energies [24,31,32]. However, using them for nanodosimetric calculations could be prohibitive in terms of simulation time and computational resources [24,25]. Thus, for dosimetric calculations in the presence of AuNPs, the low energy CH physics model Livermore was utilized using a secondary electron production cut-off energy of 100 eV. This approach seems to have good agreement with TS models that have been lately employed in nanodosimetric studies (e.g., Geant4-DNA) for target diameters ≥ 100 nm [24,25,32,33,34]. The same production energy cut value was also applied for photons. Auger electrons were activated, and the maximum electron step size was set equal to 5 nm in AuNPs clusters (half the size of the smallest cluster) and 50 nm in water (half the size of the smallest dose voxel) based on findings in the literature [35]. Moreover, in this study, these step size values were appropriate for providing accurate results combined with efficient simulation time. The histories of 2.4–3.6 × 10^9^ starting particles were simulated using the ARIS (Advanced Research Information System) HPC computational cluster, across 360 compute nodes. Statistical uncertainty was determined in each voxel and ranged from 2% to 0.4% for voxels laying 10^2^ to 10^6^ nm from the AuNPs, respectively.

### 2.2. Photon Beam Modelling

The TrueBeam^®^ Field-Dependent part was simulated according to the manufacturer’s data (Varian, Palo Alto, CA, USA). The phase space files (phsp) for both flattened and FFF photon beams used in this study were provided by the vendor. Following the manufacturer’s proposal, the 2nd version of phsp files, which contain details of particles crossing a plane above the X and Y collimators (jaws) laying 73.3 cm away from the isocenter, were used. The X and Y jaws were modeled based on the relevant information (material composition, physical density and geometrical characteristics) shared by the vendor. A water phantom of 30 × 30 × 40 cm^3^ was modeled too. In order to obtain the relevant photon field sizes at a 100 cm distance from the source, a geometrical approach was used to calculate the exact X and Y jaws positions. Intending to validate beam modeling, Percentage Depth Dose (PDD) and off-axis Dose profiles were calculated inside the mathematical water phantom. Calculations were compared against experimental data measured in a clinical TrueBeam^®^ platform. Source to water surface distance was kept constant at 100 cm, while off-axis dose measurements and calculations were performed at a depth of 10 cm inside the water phantom.

### 2.3. AuNPs Modelling

**Figure 1:**
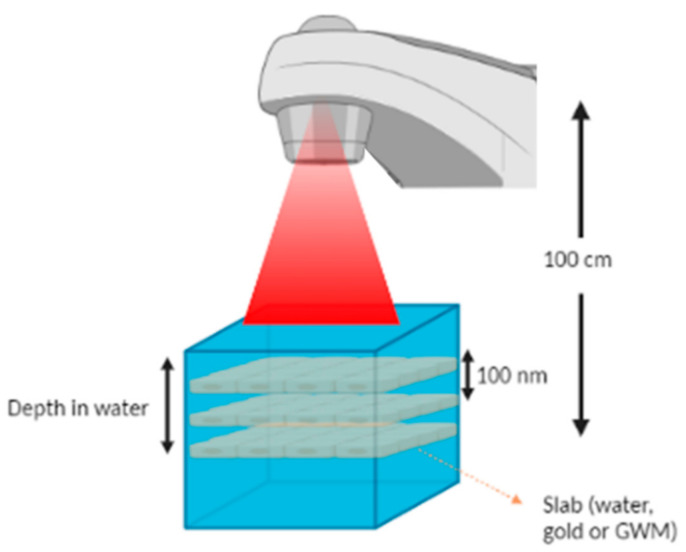
A graphical representation of the simulated irradiation geometry. The TrueBeam^®^ linac beam, the water phantom and the AuNPs clusters are depicted. Source to cluster distance (or to the last one in case of multiple clusters) was kept constant at 100 cm in all simulations. The distance between multiple clusters was set to 100 nm. Investigated depths of the slab in water were equal to 2 cm, 5 cm and 10 cm. Field sizes of 5 × 5 cm^2^ and 10 × 10 cm^2^ were examined. Created with BioRender.com.

Taking into account that AuNPs tend to form clusters once they invade cells [36,37], different types of cluster morphologies were implemented in the performed simulations. The geometry of the clusters was based on the slab model proposed by Zygmanski et al. [26]. It was assumed that AuNPs clusters can be modeled as nanometer-thin rectangular slabs containing a uniform mixture of water and gold atoms. In this model, one can use macroscopic clinical beams, while the slabs can be modeled to cover the whole cross section of the beam. The cluster thickness has the same size as that of AuNPs comprising the cluster while slab material density represents AuNPs uptake in the water slab. The geometry involved in this study is depicted in Figure 1.

### 2.4. Dosimetry Details

For each scenario, the dose was computed in water regions as a function of distance from the last AuNPs cluster (from 10^2^ to 10^6^ nm). Specifically, two different calculations for each case were performed. In the first one, the thin slab contained only water, while in the second one water was replaced with pure gold (AuSlab) or gold–water mixture (GWM). Energy deposition inside the slabs was not calculated, since it does not contribute to soft tissue dose enhancement. Dose voxel resolution was different in the range of calculations. Within the first 2 μm from the slab, voxel thickness was 100 nm while it increased to 1 μm, 10 μm and 50 μm for the ranges (2–50 μm], (50–100 μm], (100–1000 μm], respectively. The dose was reported for each voxel, while Dose Enhancement Factor (DEF) in voxel k was calculated according to:

$${\mathrm{DEF}}_{k}\;=\;\frac{D_{{\mathrm{Au}}_{k}}}{D_{w_{k}}}$$

where $D_{{\mathrm{Au}}_{k}}$ and $D_{w_{k}}$ is the deposited dose in water voxel k in presence of AuNPs and water, respectively. From depth–dose curves calculated in all simulations, DEF was averaged over different regions of interest (0–500 nm, 0–1 μm, 0–2 μm and 0–10 μm), which could include major cellular targets of ionizing radiation. Dose enhancement estimation in critical subcellular parts such as mitochondria (0.5–1 μm), cell nucleus (~1 μm) or even whole endothelial cells (~2 μm) and typical cells (~10 μm) could reveal the mechanism underlying the increased cell death that is reported in vitro. 

### 2.5. Investigated Parameters

#### 2.5.1. AuNPs Size 

Concerning AuNPs agglomeration, it seems that smaller separation distances between AuNPs are involved with greater dose enhancement [38]. In order to model the case in which AuNPs have been closely packed and trying to disengage AuNPs distribution from the dosimetric calculations [39], it was assumed that AuNPs form a thin gold slab (AuSlab), with a density of 19.3 g/cm^3^, equal to that of metallic gold. The side lengths of the slabs were 4 cm. To evaluate the effect of AuNPs size in dose increase, 10, 25, 50, 75 and 100 nm thick slabs were simulated. Calculations were repeated for both FF and FFF photon beams. Table 1 depicts the characteristics of the conducted simulations concerning the size effect in dose enhancement.

#### 2.5.2. Photon Field Size and Depth inside the Water Phantom 

For the 100 nm slab in the depth of 2 cm, 5 × 5 cm^2^ and 10 × 10 cm^2^ photon beams were used in order to examine the effect of field size on DEF. Photon fields were chosen to be greater than AuNPs clusters, to avoid the effect of photon spectrum changes in fields penumbra region. Moreover, the dose was calculated for the 100 nm AuSlab positioned at three different depths inside the water phantom (2 cm, 5 cm and 10 cm). Table 2 summarizes the irradiation characteristics that were modeled in the aforementioned evaluations.

#### 2.5.3. Concentration and Density of Clusters 

Focusing on the AuNPs concentration impact on DEF, up to three 100 nm AuSlabs were positioned at a distance of 100 nm from each other at the depth of 2 cm and were irradiated with a 5 × 5 cm^2^ photon beam in the presence and absence of FF. Assuming that AuNPs were injected in a water volume of 4 × 4 × 0.1 cm^3^, the number of one, two and three AuSlabs correspond to macroscopic concentrations of 1.9 mg/g, 3.8 mg/g and 5.7 mg/g, respectively, which are close to concentrations that have been applied on preclinical models [1,19,40]. The last part of the study represents the case where AuNPs were distributed in a 100 nm slab of water, creating a GWM of a density of 18.4 g/cm^3^ (GWMa) and 13.4 g/cm^3^ (GWMb). GWMa and GWMb contain half and 10% of the mass of gold incorporated in AuSlab, respectively. Thus, (defining *n* as the number of AuSlabs) *n*AuSlabs would contain the same mass of gold as *2n*GWMa slabs. Details about the mixture’s composition and the different investigated scenarios for AuNPs concentration in the water phantom can be found in Table 3.

## 3. Results

### 3.1. Photon Beam Model Verification

**Figure 2:**
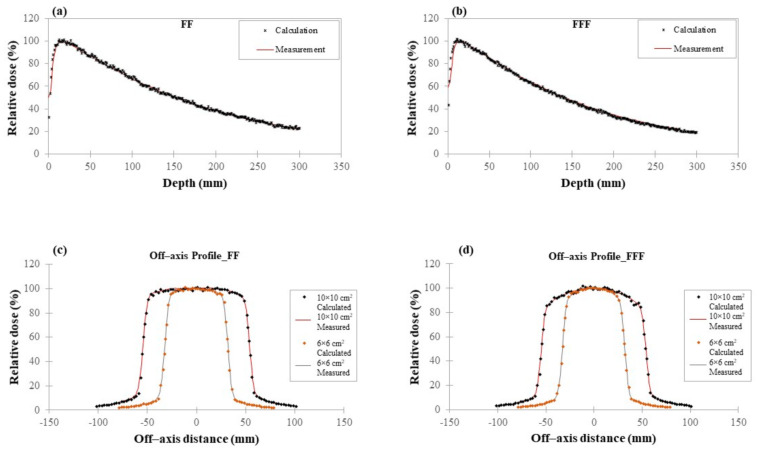
Calculated (MC simulation) and measured (clinical TrueBeam^®^ platform) relative dosimetric data: (**a**) Calculated and measured PDD comparisons for a FF 10 × 10 cm^2^ photon field; (**b**) Calculated and measured PDD comparisons for a FFF 10 × 10 cm^2^ photon field; (**c**) Off–axis profiles along left–right (LR) direction at a depth of 10 cm for a 6 × 6 cm^2^ and 10 × 10 cm^2^ FF photon field; (**d**) Off–axis profiles along LR direction at a depth of 10 cm for a 6 × 6 cm^2^ and 10 × 10 cm^2^ FFF photon field.

Indicative diagrams representing off-axis and PDD comparisons can be found in Figure 2a–d. Overall statistical uncertainties are below 2% per voxel. The average difference between calculated and measured data for any field size examined is ~2%, revealing that the used clinical photon beams were accurately modeled.

### 3.2. DEF for Different AuNPs Sizes as a Function of Distance from AuNPs Cluster

**Figure 3:**
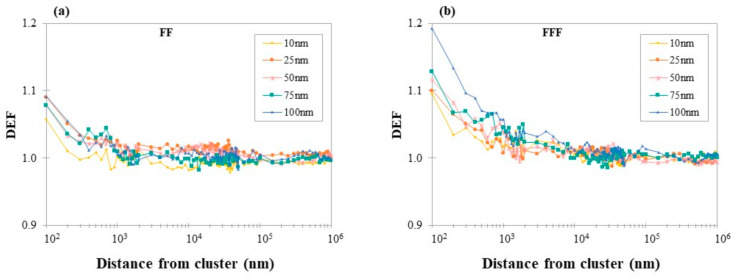
(**a**) Calculated DEF as a function of distance from different sized AuNPs clusters in case of 6 MV photon spectrum in presence of FF; (**b**) Calculated DEF as a function of distance from different sized AuNPs clusters in case of 6 MV photon spectrum in absence of FF. The X-axis is on a logarithmic scale. The photon field used to irradiate AuNPs clusters was 5 × 5 cm^2^. AuNPs clusters were positioned at a depth of 2 cm inside the mathematical water phantom. Uncertainty is below 1.6% per voxel and is not depicted. Points are connected with lines for illustration purposes.

DEF as a function of distance from AuNPs clusters for different AuNPs sizes (10, 25, 50, 75 and 100 nm) for 6 MV FF and FFF beams are shown in Figure 3a,b, respectively. DEF ranged from 0.99 ± 0.01 to 1.19 ± 0.02 depending on the presence of FF, cluster size and distance from it. As can be seen, the highest values of DEF were reported at the first 100 nm from the AuNPs cluster, in any size tested for both FF (1.09 ± 0.01) and FFF (1.19 ± 0.02) photon beams. Dose enhancement decreased with the distance from the cluster.

**Figure 4:**
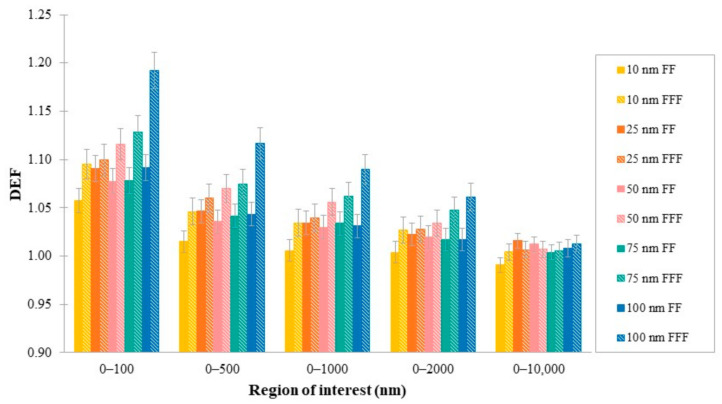
Average DEF, for different sizes of AuSlabs, in regions of interest.

DEF was averaged over distances of 500, 1000, 2000 and 10,000 nm from AuNPs clusters, corresponding to cellular targets dimensions: mitochondria (0.5–1 μm), cell nucleus (~1 μm), whole endothelial cells (~2 μm) and typical cells (~10 μm), respectively. The range 0–100 nm represents the closest distances from the AuNPs surface that were examined in this study. Average values of DEF in regions of interest are summarized in Table 4 and plotted in Figure 4 for both flattened and un-flattened photon beams. As can be seen, FFF beams produced greater levels of DEF in all studied regions and AuNPs dimensions. The differences in DEF between FFF and FF beams decreased as the distance from the cluster increased.

Near the surface of the AuNPs cluster (0–100 nm), the highest difference in dose enhancement was observed between the smallest (10 nm) and the greatest (100 nm) cluster, equal to 3% and 8% for FF and FFF beam, respectively. Moving away from the cluster’s surface, DEF differences due to the AuNPs size decreased. Examining targets of interest, in the region of 0–500 nm, the average DEF difference between 10 and 100 nm was found to be equal to 3% for the FF and 7% for the FFF photon beams falling to 2% and 6%, respectively, in the region 0–1 μm. At distances up to 2 μm, average DEF for the 100 nm AuSlab was found to be 2% (FF) and 3% (FFF) greater than that of 10 nm. Average DEF in targets 0–10 μm appeared to be independent of AuNPs cluster size.

### 3.3. DEF as a Function of Number of AuSlabs

**Figure 5:**
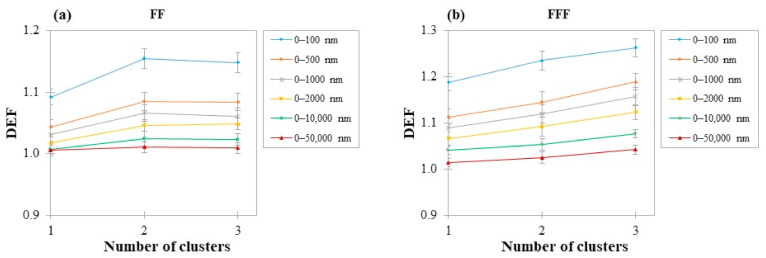
(**a**) Comparison of DEF among regions of interest for different numbers of AuSlabs in the case of 6 MV photon spectrum in presence of FF; (**b**) Comparison of DEF among regions of interest for different numbers of AuSlabs in the case of 6 MV photon spectrum in absence of FF. The size of all examined clusters was 100 nm and the photon field used to irradiate AuNPs clusters was 5 × 5 cm^2^. AuNPs clusters were positioned at a depth of 2 cm inside the mathematical water phantom. Points are connected with lines for illustration purposes.

The impact of increasing the number of AuNPs clusters in a water medium was investigated. Figure 5 depicts the average DEF for different regions for all AuSlabs investigated. For more than one AuSlab, average DEF ranged from 1.01 ± 0.01 to 1.15 ± 0.02 and from 1.01 ± 0.01 to 1.26 ± 0.02 for FF and FFF beams, respectively, depending on the target distance from the last slab. While in FF photon beams, adding more than two slabs had a low impact on calculated DEF, in the case of FFF beams, an almost linear relationship between the number of clusters and DEF was observed. 

When the number of clusters was doubled, in the first 100 nm from AuSlabs, DEF increased by 6% and 4% for FF and FFF beams, respectively. In a region of 0–500 nm from the last cluster, the corresponding increase in DEF was 4% and 3% for FF and FFF photon beams, respectively. For targets 0–1 μm average DEF increased by 3% for both photon spectrums. In the region 0–2 μm a 3% and 2% increase in DEF was found. The average DEF increase in the range of 0–10 μm was 1% (FF) and 2% (FFF).

From one to three slabs, average DEF in the range of 0–100 nm increased by 5% and 6% for FF and FFF beams, respectively. In the rest regions of interest, for FF photon beams, the DEF increase was the same as that noticed in the case of doubling the number of clusters. For FFF photon beams, a 7%, 6%, 5% and 3% raise in DEF was found in targets 0–500 nm, 0–1 μm, 0–2 μm and 0–10 μm, respectively.

### 3.4. DEF as a Function of GWM Density

**Figure 6:**
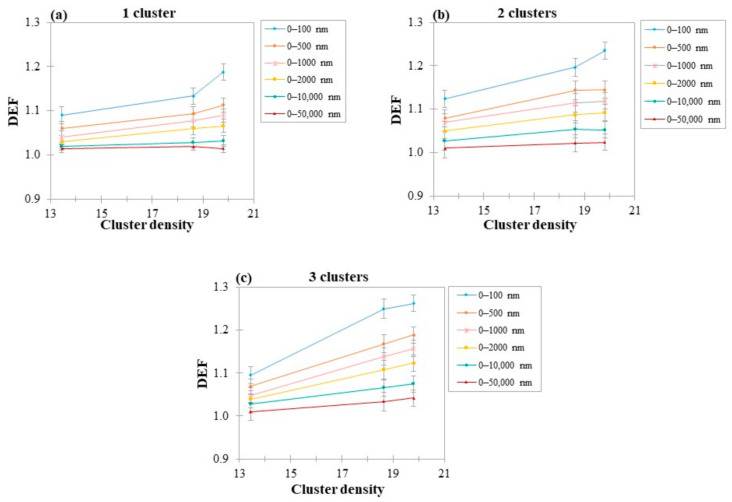
(**a**) DEF as a function of slab density for 1 cluster; (**b**) DEF as a function of slab density for 2 clusters; (**c**) DEF as a function of slab density for 3 clusters. The size of all examined clusters was 100 nm and the photon field used to irradiate AuNPs clusters was 5 × 5 cm^2^ (FFF). AuNPs clusters were positioned at a depth of 2 cm inside the mathematical water phantom. Points are connected with lines for illustration purposes.

The simulations were repeated for two and three GWMa (18.3 g/cm^3^) and GWMb (13.4 g/cm^3^) slabs (Table 3). Results are presented in Figure 6, showing that as GWM density increased, DEF increased also for almost all regions of interest. AuSlab exhibited the highest values of DEF, while for the GWMb slab, which has the lowest concentration of gold, dose enhancement did not exceed 12% in any scenario. The differences in DEF between the lowest and highest densities increased with the number of clusters.

**Figure 7:**
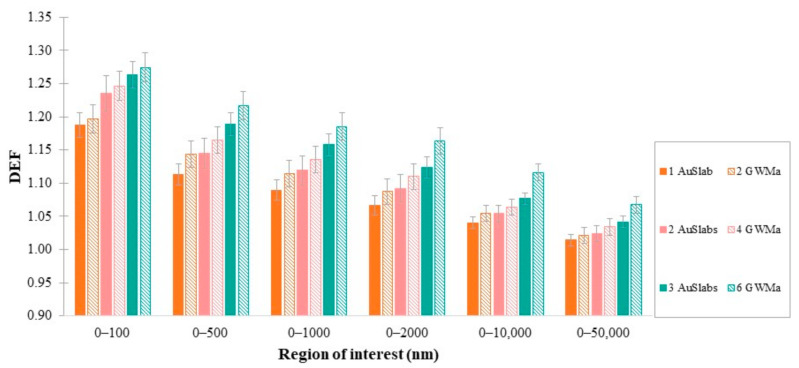
Comparison between different arrangements of the same mass of AuNPs. The size of all examined clusters was 100 nm, and the photon field used to irradiate AuNPs clusters was 5 × 5 cm^2^ (FFF). AuNPs clusters were positioned at a depth of 2 cm inside the mathematical water phantom.

As can be seen from Table 3, the mass of gold in the case of one AuSlab equals the mass of gold in two GWMa slabs. Therefore, *n*AuSlabs contain the same mass of gold as 2*n*GWMa slabs. Figure 7 shows the comparison between the calculated average DEF when the same mass of gold was added to the water phantom in two different patterns. In any region, for the same mass of gold incorporated in water, the arrangement of 2*n*GWMa slabs tended to produce higher dose enhancement than the corresponding one with *n*AuSlabs, where *n* is the number of AuSlabs (*n* = 1, 2, 3). 

**Figure 8:**
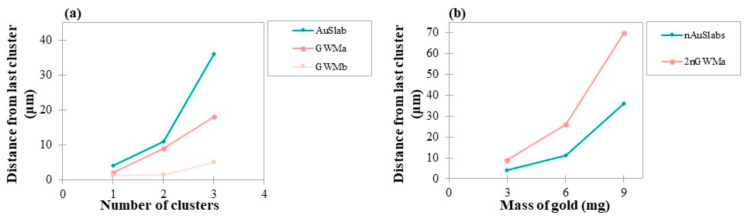
(**a**) Distance from AuNPs clusters where DEF remains ≥1.04 as a function of clusters number. AuSlab, GWMa and GWMb have densities of 19.3 g/cm^3^, 18.43 g/cm^3^ and 13.4 g/cm^3^ and contain 3, 1.5 and 0.3 mg of gold, respectively; (**b**) Distance from AuNPs clusters where DEF remains ≥1.04 as a function of the mass of gold. Results are depicted for *n* = 1, 2 and 3 where *n* is the number of AuSlabs. The corresponding mass of gold embedded in the water phantom for *n* = 1, 2 and 3 is equal to 3 mg, 6 mg and 9 mg, respectively. Points are connected with lines for illustration purposes.

In order to discuss the obtained findings related to the range of dose enhancement from multiple clusters of different densities, the distance from the AuNPs surface in which DEF values were greater or equal to 1.04 ± 0.01 was examined. Depending on the number of clusters, ranges of 4–36 μm, 2–70 μm and 1.2–5 μm were reported in the case of AuSlab, GWMa and GWMb slabs, respectively. Figure 8a provides range comparisons between clusters of different densities. In Figure 8b, the range of dose enhancement is illustrated as a function of the mass of gold for the two clusters arrangements (*n*AuSlabs–2*n*GWMa). For *n* = 1, 2 and 3 the corresponding mass of gold is equal to 3 mg, 6 mg and 9 mg, respectively.

### 3.5. DEF as a Function of Photon Field Size and Depth of AuNPs Cluster in Water 

**Figure 9:**
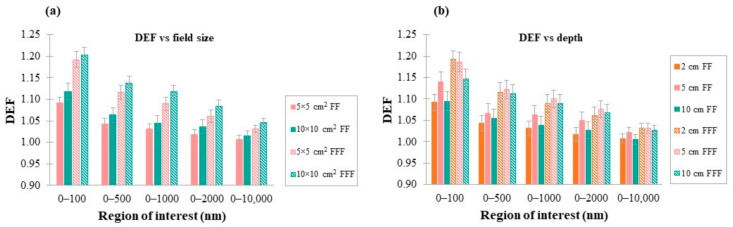
(**a**) Average DEF in regions of interest for two field sizes inside the mathematical water phantom. AuSlab was positioned at a depth of 2 cm inside the mathematical water phantom; (**b**) Average DEF in regions of interest for the different depths inside the mathematical water phantom. The size of AuSlab was 100 nm and the photon field used was 5 × 5 cm^2^.

The effect of photon field size was investigated for a 5 × 5 cm^2^ and a 10 × 10 cm^2^ photon field corresponding to both FF and FFF spectrums (the 100 nm AuSlab was positioned at 2 cm in water) and the results are presented in Figure 9a. Doubling photon field size from 5 × 5 cm^2^ to 10 × 10 cm^2^ was translated to a 1–2% and 1–3% increase in average DEF for FF and FFF photon fields, respectively, depending on the region of interest.

DEF was calculated for the 100 nm AuSlab positioned at 2 cm, 5 cm and 10 cm depth inside the water phantom. Field size was kept constant at 5 × 5 cm^2^ (source to cluster distance = 100 cm). Results from simulations for both FF and FFF photon beams are presented in Figure 9b. For FF photon beams, DEF at 5 cm was 1–4% greater than 2 cm and 10 cm, while for FFF beams, the maximum reported difference was 1%. DEF at 10 cm was 1% greater than 2 cm for both photon spectrums. The only exception was the region 0–100 nm in FFF photon beam irradiation, where DEF at 2 cm was 4% greater than that in the case of 10 cm. 

## 4. Discussion

In the present study, the impact of AuNPs properties and photon beam characteristics on dose enhancement as a function of distance from AuNPs was evaluated. 

### 4.1. Dependence of DEF on FF 

In all examined scenarios, FFF beams led to higher DEF values, a finding that is consistent with in vitro [28] and in silico [4,13,14] findings of the peer-reviewed literature (Figure 3, Figure 4, Figure 5 and Figure 9 and Table 4). These outcomes can be explained by the softer photon energy spectrum, which contains a greater amount of low-energy photons compared to the corresponding spectrum of flattened photon beams. Low-energy photons in beams can increase photoelectric interactions with AuNPs and, consequently, enhance dose deposition in water.

### 4.2. DEF as a Function of Distance from AuNPs Clusters

Dose enhancement was found to decrease with distance from the AuNPs surface as seen in Figure 3. In all studied scenarios, the greatest dose enhancement was found at a distance of 0–100 nm from AuNPs clusters. This finding is supported by the fact that the range of most Auger e^–^, which are produced following ionizing events around AuNPs clusters, have low energy and a short range in water (~200 nm) [41]. In this range, the dose could be enhanced by up to 27%, depending on the number of AuSlabs, their size and the beam quality. This outcome depicts that ifAuNPs accumulate close to subcellular parts of 100 nm, dose deposition in these targets could be sufficiently increased, which is in good accordance with the literature data [4,42]. 

In flattened photon beams, the highest DEF in the first 100 nm from one AuSlab was found equal to 1.09 ± 0.01, which is in agreement with corresponding results published in the literature [11,16]. DEF fell rapidly to 1.02 ± 0.01 at 1 μm away from the AuSlab surface (Figure 3a). In the case of un-flattened photon beams irradiation, the greatest dose enhancement was found at 1.19 ± 0.01 dropping to 1.02 ± 0.01 at distances of up to 8–10 μm from AuSlab (Figure 3b).

The distance from the cluster upon which a DEF ≥ 1.04 can be observed in FFF photon beam irradiation seems to depend mainly on the number of clusters inside the water phantom. As seen in Figure 8a, this distance increased with the cluster’s density and the number of clusters, probably due to the increased number of photon–AuNPs interactions and secondary electrons produced. According to Figure 8b, for the scenarios involving the same mass of gold, the arrangement of *2n*GWMa slabs doubled the range where DEF ≥ 1.04 was found, compared to *n*AuSlabs. Owing to the phenomenon of self-absorption [43,44], the produced secondary e^–^ are more likely to escape from a less dense material such as GWMa. Since they suffer from small energy losses inside GWMa, they would deposit their energy at greater distances from clusters.

### 4.3. Dependence of DEF on AuNPs Size 

Concerning AuNPs size, the obtained results depict that cluster size was weakly related to DEF in flattened MV photon beam irradiation (Figure 3a), which is in close agreement with published in silico studies [14,16,17]. This finding could imply that the increased photon interactions with AuNPs of greater size (increased number of gold atoms) could not compensate for the self-absorption of the produced secondary e^–^. MC studies with 6 MV-flattened photon beams report deviations <1% in average DEF between the examined sizes [14,16,17], while in others, no difference is mentioned [10,15]. Recently, a poor relationship between the biological effects of irradiated AuNPs clusters and their size has been exhibited [45]. To the authors’ knowledge, a limited number of computational studies have reported a size impact on DEF under flattened photon beam irradiation [4,11]. For instance, comparing AuNPs of 10 and 100 nm for various clinical cases, Tsiamas et al. [4] suggested that 100 nm could be more efficient, especially in out-of-field areas and for greater field size. Keshavarz et al. [11] who modeled X-ray spectra generated by a 4 MV linac, reported that the DEF of 25 nm and 100 nm AuNPs differs by ~2.8%, which was the highest stated difference. 

In the case of FFF beams, size impact on DEF remained moderate but was more pronounced compared to flattened beams (Figure 3b). This finding could be attributed to the softer photon spectrum of FFF photon beams compared to flattened, which increases the probability of photon interactions with AuNPs of larger sizes. The maximum DEF was found at 1.19 for the 100 nm AuSlab, which is 8.2% greater than the corresponding value for 10 (or 25 nm). This was the greatest difference between sizes and was found in the first 100 nm from AuSlabs, indicating a size–DEF relationship at the closest distances to AuNPs, which is consistent with the literature [4,41]. Indeed, it has been shown that AuNPs size will dictate whether Auger e^–^ will stop inside gold or escape in the surrounding water regions [44]. In the latter case, due to their short range, Auger e^–^ will deposit their energy adjacent to the AuNPs’ surface at distances < 1 μm. The highest average DEF in all regions of interest was calculated for the 100 nm AuNPs cluster, which is in agreement with similar conclusions [4]. 

It should be noted though that several in vitro studies noticed that the radiosensitization effect on cells increases with the size of AuNPs [20,46,47], while at the same time, larger AuNPs have been correlated to increased tissue toxicities [46]. Guo et al. [20] have found higher AuNPs uptake in the case of 30.5 vs. 14.4 nm followed by an 8% increase in liver cells radiosensitization. Outcomes from the irradiation of MDA-MB-231 cells with a 6 MV X-ray beam suggest higher radiosensitivity in the case of 49 nm AuNPs in comparison to 16 nm (sensitivity enhancement ratio of 1.86 and 1.49, respectively) [46]. Rahman et al. [47] showed a greater reduction in HeLa cell survival using 15 nm AuNPs compared to 5 nm when irradiated with a 6 MV X-ray beam. On the other hand, having performed gel dosimetry, Behrouzkia et al. [48] found the highest DEF (1.17, 1.12 and 1.10) using 50 nm AuNPs, followed by 100 nm and 30 nm. 

### 4.4. Dependence of DEF on Clusters Density 

With regard to cluster density, it was found that higher density leads to higher DEF values (Figure 6). This could be expected given the fact that higher density implies that a greater number of AuNPs have been distributed in the water slab. Hence, since photon–AuNPs interactions increased, dose deposition was amplified. 

### 4.5. Dependence of DEF on the Number of Clusters

As seen in Figure 5, adding more than one cluster inside the water phantom appears to have a greater impact on DEF in the shortest distances from AuNPs. In general, examining distances from AuNPs clusters up to 10 μm, when the number of AuSlabs was doubled and tripled, average DEF increased by 2–5% and 3–7%, respectively, depending on the target region and the presence of FF. Published MC studies for MV photon beams demonstrate a 1–6% increase in DEF when macroscopic concentration is tripled, which is close to our results [14,17,49], while in vitro, there has been noticed a greater increase in radiosensitivity with concentration [27,50]. 

In this work in the presence of FF, no average DEF difference was found between two and three AuSlabs (Figure 5). It could be implied that in the case of FF beam irradiation, part of the secondary e^–^ produced from photon interactions with the first AuSlabs was absorbed by AuSlabs lying underneath. Thus, adding more than two slabs increased the absorption of secondary e^–^, preventing dose deposition outside AuNPs. On the contrary, for the un-flattened photon beams, a 2–4% average DEF increase was found between two and three AuSlabs. This outcome could be explained considering that in un-flattened photon beams irradiation, absorption of short-range e^–^ from multiple AuNPs clusters could be counterbalanced by the increased number of secondary e^–^ produced due to the softer FFF photon beam spectrum. This study suggests that the highest dose enhancement differences with concentration, arise in the case of FFF beams, similar to the literature [14,51], and at close distances to clusters.

For the examined AuNP concentrations and photon spectrums, at any distance up to 1 mm from AuNPs clusters, photon beam attenuation attributed to the presence of multiple AuNPs clusters did not exceed 1% within statistical uncertainty. In their study though, Chow et al. [51] concluded that DEF decreased when the concentration of AuNPs increased in a water phantom reaching values <1. These outcomes were associated with the phenomenon of photon beam attenuation due to the presence of gold, which was proven to be more important for higher AuNP concentrations in certain depths. 

In the case of GWMa slab irradiation (FFF photon beam), average dose enhancement at distances up to 10 μm increased by 3–6% and 4–10% when the number of slabs was doubled and tripled, respectively (Figure 6). The obtained differences were higher than the corresponding in the case of AuSlabs (2–4% and 3–7%). As mentioned above, this outcome could be associated with the phenomenon of self-absorption [43,44], which is possibly more prominent in the dense AuSlab than in the GWMa slab. However, for GWMb, which has a lower density, slabs increase was translated to a 1–3% increase in average DEF.

### 4.6. Dependence of DEF on Clusters Modelling

Based on the assumption that AuSlab represents a scenario of densely packed AuNPs, while GWM slabs are correlated to homogeneous mixtures of water and gold, DEF values when the same mass of gold was embedded in a water phantom were compared. It can be seen in Figure 7 that for the same mass (*n*AuSlabs vs. *2n*GWMa slabs) in any region of interest, the concept of 2*n*GWMa slabs produced moderately higher DEFs than the scenario of *n*AuSlabs (for *n* = 1, 2, 3). Throughout the first 10 μm from clusters, the greatest difference between the two arrangements was found for the greatest mass of gold inside the phantom (~5%). Results from this work indicate that the distribution of AuNPs inside a tumor region could be of critical importance for both dose enhancement and the range that it is noticed. AuNPs uptake from cells and the pattern of diffusion should be thoroughly investigated since they might be of critical importance for assessing the levels of dose enhancement and possible “organs at risk” toxicities.

### 4.7. Dependence of DEF on Photon Field Size and Depth of AuNPs in Water

As presented in Figure 9a, increasing photon field size from 5 × 5 to 10 × 10 cm^2^ was translated to a 1–3% increase in DEF. It is important to note that TrueBeam^®^ head components (e.g., multileaf collimator, baseplate, etc.) have not been implemented in the conducted simulations. Thus, scatter radiation from beam interactions with linac head modules has not contributed to the calculated dose distributions. The lower energy of the scatter component of the photon beam compared to the primary one, could slightly increase DEF due to the increased photons—gold interaction probability in this energy range.

The scatter component of the photon beam increases with depth in water (up to a certain depth beyond which, it slowly decreases). Supposing that scattered particles arising from photon beam interactions with the water lying above AuNPs clusters, due to their lower energy, could lead to increased dose enhancement at greater depths, the relation between the depths of AuNPs in water (2 cm, 5 cm and 10 cm) and DEF was investigated. As displayed in Figure 9b, DEF increased with depth up to 5 cm and decreased at 10 cm. At the depth of 2 cm, low-energy photons of the beam might reach AuNPs and interact with them, leading to sufficient dose enhancement. As depth increases to 5 cm, the photon spectrum could probably be softer due to the increase in the Compton scatter component. At the depth of 10 cm, attenuation of low-energy photons in water could affect the increasing trend of the scatter component. This phenomenon might limit the number of low-energy photons that will reach AuNPs and, therefore, restrict dose enhancement. The effect of depth could probably be more important in out of central axis areas or for greater photon field sizes [4,13].

### 4.8. Average DEF in Cellular Regions of Interest

In this study, sufficient dose enhancement was calculated in regions that represent important cellular regions or include subcellular targets depending on the aforementioned parameters. In cases where AuNPs accumulate inside the cell, dose increase could reach all parts of an endothelial (~2 μm) or typical cell (~10 μm), since average DEFs range from 1 ± 0.01 to 1.16 ± 0.02 and 0.99 ± 0.01 to 1.12 ± 0.02, respectively. Substantial DEF values could be observed when AuNPs approach mitochondria (0.5–1 μm) and the cell nucleus (~1 μm). Depending on their size, mitochondrial DEF could reach 1.22 ± 0.02. Nucleus DEF could range from 1.01 ± 0.01 to 1.19 ± 0.02.

### 4.9. Future Prospects

While great progress has been achieved in AuNP-enhanced RT research, there exist limitations complicating the attempts for clinical translation. Interdisciplinary research is needed in order to define the ideal size—concentration combination that maximizes AuNPs uptake from cells and tumor control probability, followed by limited toxic effects. Moreover, attention should be paid to photon beam attenuation due to the presence of high concentrations of AuNPs in an irradiated volume, and the balance between it and dose enhancement [51]. Future research should also encompass biological models in MC calculations so that the outcomes concerning dose deposition can be associated with the corresponding cellular damages. The reported differences in the radiosensitivity effect between in vitro studies and theoretical models may be attributed to the biological mechanism that possibly drives AuNPs RT with MV photons. The increased levels of oxidative stress and reactive oxygen species (ROS) resulting from AuNPs presence in irradiated tissues are considered key parameters that could probably affect the enhancement of tumor cell destruction that is noticed in vitro [23,46,52]. Finally, the experimental verification of MC findings could further explain the differences that have been reported between in silico and in vitro studies. Aiming in precise cross-examination, more realistic models should be used related to AuNPs’ ideal physicochemical characteristics, dosage and biodistribution pattern.

## 5. Conclusions

In this study, the irradiation of AuNPs clusters positioned in a mathematical water phantom was modeled. Dose enhancement was estimated as a function of distance from AuNPs for different clinical scenarios. A sufficient dose amplification was found while the ranges where DEF is meaningful represent important cellular targets (endothelial or typical cells) and subcellular regions (mitochondria or nucleus). Depending on AuNPs localization inside a cell, different levels of dose enhancement could be achieved. Therefore, it can be concluded that 6 MV FFF photon fields irradiation of AuNP (size of 100 nm)-loaded regions may increase the deposited dose, with the greatest effect on close distances from clustering geometries. 

## Figures and Tables

**Table 1:** Simulation parameters for size effect in dose enhancement.

AuSlab Size (nm)	Field Size	Depth	Photon Beam
10	5 × 5 cm^2^	2 cm	FF, FFF
25			
50			
75			
100			

**Table 2:** Simulation parameters for field size and depth in water impact on dose enhancement.

Field Size (cm^2^)	Depth (cm)	Photon Beam	AuSlab Size
5 × 5	2, 5, 10	FF, FFF	100 nm
10 × 10	2		

**Table 3:** Simulated 100 nm clusters characteristics and irradiation conditions for different concentrations. Photon field size was 5 × 5 cm^2^, and the depth in water for the last slab was 2 cm.

Cluster ID	Concentration by Mass (%)		Au Mass (mg)	Density (g/cm^3^)	Photon Beam	Number of Clusters
	Water	Au				
AuSlab	0	100	3	19.3	FF, FFF	1, 2, 3
GWMa	5	95	1.5	18.4	FFF	1, 2, 3, 4, 6
GWMb	68	32	0.3	13.4	FFF	1, 2, 3

**Table 4:** Average values of DEF with the calculated errors in regions of interest.

Cluster Size (nm)	Region of Interest (nm)				
	0–100	0–500	0–1000	0–2000	0–10,000
	FF				
10	1.06 ± 0.01	1.01 ± 0.01	1.01 ± 0.01	1 ± 0.01	0.99 ± 0.01
25	1.09 ± 0.01	1.05 ± 0.01	1.03 ± 0.01	1.02 ± 0.01	1.02 ± 0.01
50	1.08 ± 0.01	1.04 ± 0.01	1.03 ± 0.01	1.02 ± 0.01	1.01 ± 0.01
75	1.08 ± 0.01	1.04 ± 0.01	1.03 ± 0.01	1.02 ± 0.01	1 ± 0.01
100	1.09 ± 0.01	1.04 ± 0.01	1.03 ± 0.01	1.02 ± 0.01	1.01 ± 0.01
	FFF				
10	1.10 ± 0.02	1.05 ± 0.01	1.03 ± 0.01	1.03 ± 0.01	1.02 ± 0.01
25	1.10 ± 0.02	1.06 ± 0.01	1.04 ± 0.01	1.03 ± 0.01	1.01 ± 0.01
50	1.12 ± 0.02	1.07 ± 0.01	1.06 ± 0.01	1.03 ± 0.01	1.02 ± 0.01
75	1.13 ± 0.02	1.07 ± 0.01	1.06 ± 0.01	1.05 ± 0.01	1.02 ± 0.01
100	1.19 ± 0.02	1.12 ± 0.02	1.09 ± 0.02	1.06 ± 0.01	1.03 ± 0.01

## Data Availability

All data generated or analyzed during this study are included in this published article.
